# Wnt5a in keratinocytes contributes to complex regional pain syndrome through the activation of NR2B and MMP9 in rats

**DOI:** 10.1136/rapm-2024-106139

**Published:** 2025-03-12

**Authors:** He Zhu, Bei Wen, Jijun Xu, Li Xu, Yuguang Huang

**Affiliations:** 1 Department of Anesthesiology, Peking Union Medical College Hospital,Chinese Academy of Medical Sciences & Peking Union Medical College, Beijing, China; 2 Department of Pain Management, Cleveland Clinic, Cleveland, Ohio, USA

**Keywords:** Animal Experimentation, CHRONIC PAIN, Pain Management, Complex Regional Pain Syndromes

## Abstract

**Background:**

Complex regional pain syndrome (CRPS) is a chronic pain condition characterized by inflammatory features, though the underlying mechanisms remain partly understood. Our study examined whether Wnt5a in skin keratinocytes contributes to CRPS-related pain hypersensitivity by activating downstream N-methyl-D-aspartate receptor subunit 2B (NR2B) and matrix metalloproteinase-9 (MMP9) signaling in rats.

**Methods:**

We developed a cell-culture model to mimic the local inflammation of CRPS and a rat model to mimic the chronic post-ischemia pain experienced by CRPS patients. Mechanical and heat pain thresholds in the hind paw were measured using an electronic von Frey apparatus and a radiant heat device. Western blotting and immunofluorescence were used to examine the expressions of NR2B and MMP9 in the skin and dorsal root ganglion (DRG), and immunofluorescence staining of connexin 43 (Cx43) and protein gene product 9.5 (PGP9.5) were conducted to explore the interaction between keratinocytes and nerve fibers in the skin.

**Results:**

In cell culture, Wnt5a was expressed in keratinocytes and contributed to cellular injury by increasing the levels of NR2B and MMP9. The mechanical and heat pain thresholds measured in the hind paw were decreased in CRPS rats, indicating increased pain sensitivity. The inhibition of Wnt5a alleviated these CRPS-related pain hypersensitivities. High levels of Cx43 and PGP9.5 staining were observed in the epidermis of CRPS rats, suggesting an interaction between keratinocytes and nerve fibers that may contribute to CRPS. Additionally, upregulations of NR2B and MMP9 in the DRG may further exacerbate pain.

**Conclusions:**

Skin keratinocytes may play an essential role in the pathophysiology of CRPS. Wnt5a signaling may increase pain sensitivity by upregulating downstream NR2B and MMP9, thereby contributing to CRPS.

**WHAT IS ALREADY KNOWN ON THIS TOPIC**

- The identification of new therapeutic targets for complex regional pain syndrome (CRPS) is important due to the current lack of effective treatments. Previous studies have highlighted the potential role of Wnt5a in the pathophysiology of chronic pain. However, it remains unclear whether Wnt5a is expressed in skin keratinocytes and involved in CRPS.

**WHAT THIS STUDY ADDS**

- Our study demonstrates that Wnt5a in skin keratinocytes is involved in CRPS-related pain by activating downstream NR2B and MMP9. The interaction between keratinocytes and nerve fibers provides new insight into the pathogenesis of CRPS.

**HOW THIS STUDY MIGHT AFFECT RESEARCH, PRACTICE OR POLICY**

- Our findings reveal an essential role of Wnt5a in keratinocytes in CRPS pathogenesis and suggest that the application of Wnt5a inhibitors to the skin may alleviate CRPS.

## Introduction

 Complex regional pain syndrome (CRPS) is a chronic pain condition with a prevalence of approximately 5.4–26.2 per 100 000 person-years, and it occurs acutely in 7% of patients with limb injuries.^1 2^ Peripheral neuron sensitization contributes to the clinical symptoms of CRPS, including spontaneous pain, hyperalgesia, and allodynia. Over the past 30 years, research into CRPS has expanded extensively, revealing that persistent regional inflammation promotes pain sensitization.^3^

Skin keratinocytes can elicit pain through the activation of transient receptor potential (TRP) channels on sensory neurons and the release of neuroactive mediators when inflammation and injury occur.^4^ The nerve fibers in the dermis and epidermis contribute to a wide variety of sensory functions. Intriguingly, a recent study suggested that the interaction of keratinocytes and nerve fibers in the skin may have substantial implications for chronic pain.^5^

Previous studies have also highlighted the significant role of Wnt5a, a representative noncanonical Wnt ligand, in promoting peripheral neuropathic pain in rodents.^6 7^ Single-cell analysis of psoriasis demonstrates Wnt5a positive (^+^) fibroblasts upregulate multiple inflammatory genes in keratinocyte hyperplasia.^8^ However, it remains unclear whether Wnt5a in keratinocytes is involved in CRPS.

Our recent study demonstrated that the N-methyl-D-aspartate receptor subunit 2B (NR2B) plays a pivotal role in CRPS through oxidative stress and inflammation.^9 10^ Wnt5a/NR2B signaling has been suggested to regulate neuropathic pain.^11 12^ Additionally, matrix metalloproteinase 9 (MMP9) has been shown to contribute to the development and maintenance of neuropathic pain.^13^ Bioinformatics analysis based on blood samples from CRPS patients has also identified MMP9 as a potential target for alleviating CRPS.^14^ Taken together, these findings suggest that NR2B and MMP9 may be new therapeutic targets for CRPS.

Currently, there is a lack of effective treatments for CRPS. Previous studies have primarily focused on keratinocytes as a physical and chemical barrier for the human body. In this pre-clinical study, we aimed to determine the role of keratinocytes and Wnt5a in CRPS and provide new insights into the molecular pathogenesis of this chronic pain condition.

## Methods

### Animals

Adult male Sprague-Dawley rats were housed under standard conditions at the Experimental Animal Center of Peking Union Medical College Hospital (PUMCH; Beijing, China). The human keratinocyte cell line (HaCaT cells) used for RNA-Seq analysis was obtained from China Infrastructure of Cell Line Resource (Beijing, China). The study was reported in accordance with the guidelines of Animal Research: Reporting of In Vivo Experiments.^15^

### Primary keratinocyte isolation and culture

Keratinocytes, the predominant cell type in the epidermis, were isolated from rats as described previously.^16 17^ Briefly, postnatal day 0–2 neonates from the Sprague-Dawley rats were sacrificed by decapitation. The whole skin was peeled off, and the epidermis was separated using 0.3% Dispase II. The epidermis was treated with 0.05% Trypsin-EDTA for 20 min at 37*°*C to obtain keratinocytes (online supplemental figure S1).

### Experimental protocol

In the in vitro cell experiment, keratinocytes were divided into four groups: Control, Oxygen–glucose deprivation/reoxygenation (OGD/R), shRNA-NC (shRNA negative control transfection), and shRNA-102 (shRNA Wnt5a transfection). The OGD/R, shRNA-NC, and shRNA-102 groups were then treated with OGD/R. Glucose-free MEM (Pricella, Wuhan, China) was used to induce OGD for 3 hours under low-oxygen conditions with MGC AnaeroPack (Mitsubishi Gas Chemical Co., Japan).

In the in vivo animal experiments, rats were randomly assigned to the Sham and Chronic postischemia pain (CPIP) groups. The CPIP group was further divided into three treatment subgroups: CPIP (100 µL normal saline), Foxy-5 (a Wnt5a agonist, 50 µmol/L, 100 µL), and Box5 (a Wnt5a inhibitor, 100 µmol/L, 100 µL). The drugs were injected into the ventral side of the hind paw once a day from day 8 to day 14 before tissue extraction. The CPIP modeling and drug administration flow chart are shown in online supplemental figure S2.

### Knocking down Wnt5a by shRNA transfection

Lentiviral vectors containing shRNA-Wnt5a (shRNA102, 672 and 1022) and shRNA-NC were designed and produced by Genomeditech (Shanghai, China). When the density of keratinocytes reached 70%, they were transfected with lentiviral vectors containing shRNA Wnt5a or shRNA-NC following the manufacturer’s instructions. The details are presented in online supplemental table S1.

### Behavioral testing

Mechanical withdrawal threshold (MWT) was assessed using a calibrated electronic von Frey apparatus. Thermal withdrawal latency (TWL) was assessed using a radiant heat device. Stimulation was applied to the plantar surface of the ipsilateral hind paw, with repeated measurements taken three times at approximately 10 min intervals.^9^

### Real-time quantitative PCR

The total RNA of keratinocytes was extracted and reverse transcribed into cDNA using an RNA extraction kit (HaiGene, Harbin, China) and Prime Script RT Master Mix (RR036; Takara, Otsu, Japan). Quantitative PCRs (qPCRs) were performed using SYBR Premix Ex Taq (Takara, Osaka, Japan) and a StepOne Real-Time PCR System (Applied Biosystems, Foster City, California, USA). Primers for the selected genes are shown in online supplemental table S2.

### Western blotting and immunofluorescence

The protein samples of keratinocytes and rat tissues were prepared as described previously.^9^ The protein samples were then separated by 10% SDS-PAGE and transferred to polyvinylidene fluoride membranes. After 2 hours of blocking with 5% skim milk, the membranes were incubated in primary antibodies at 4°C overnight. The blots were washed and further incubated with HRP-conjugated secondary antibody for 2 hours.

For immunofluorescence, the cells and tissues were prepared as described previously.^10^ The L4–L5 dorsal root ganglions (DRGs) were separated after paraformaldehyde fixation. The paraffined DRG tissues were then cut into 10 µm thick sections and permeabilized with 0.3% Triton X-100 for 10 min. The sections were then blocked with 5% normal goat serum for 1 hour and incubated with primary antibodies overnight at 4°C. The next day, the secondary antibodies were added for 2 hours and then sealed with an antifade solution containing DAPI. The antibodies used are shown in online supplemental table S3.

### Measurement of mitochondrial membrane potential and the reactive oxygen species analysis

Changes in the mitochondrial membrane potential were assessed using a JC-1 assay kit (C2006; Beyotime, Shanghai, China). Mitochondrial membrane depolarization was monitored by changes in the red/green fluorescence ratio, where a decreased ratio indicates decreased mitochondrial membrane potential.^18^ The reactive oxygen species (ROS) Assay Kit (S0033S, Beyotime, China) was used to analyze intracellular ROS levels.^10^

### RNA-sequencing analysis

Human keratinocytes were used for RNA-seq analysis to identify the hub gene related to CRPS. Differentially expressed genes (DEGs) were uploaded to the STRING database, and the protein–protein interaction (PPI) network map was visualized using Cytoscape software V.3.9.1. Gene Set Enrichment Analyses (GSEA) enrichments were estimated using the normalized enrichment score (NES).

### Statistical analysis

A two-tailed Student’s t-test was used to compare differences between two groups, whereas one-way analysis of variance (ANOVA) was used for comparisons among three or more groups. Behavioral data were analyzed using two-way repeated-measures ANOVA. Post hoc tests with Bonferroni correction were applied for multiple comparisons. Data were analyzed using SPSS V.23.0 software (SPSS). Data are presented as mean±SEM, and a p<0.05 was considered significant.

## Results

### Expression of Wnt5a in primary keratinocytes and rats after modeling

**Figure 1:**
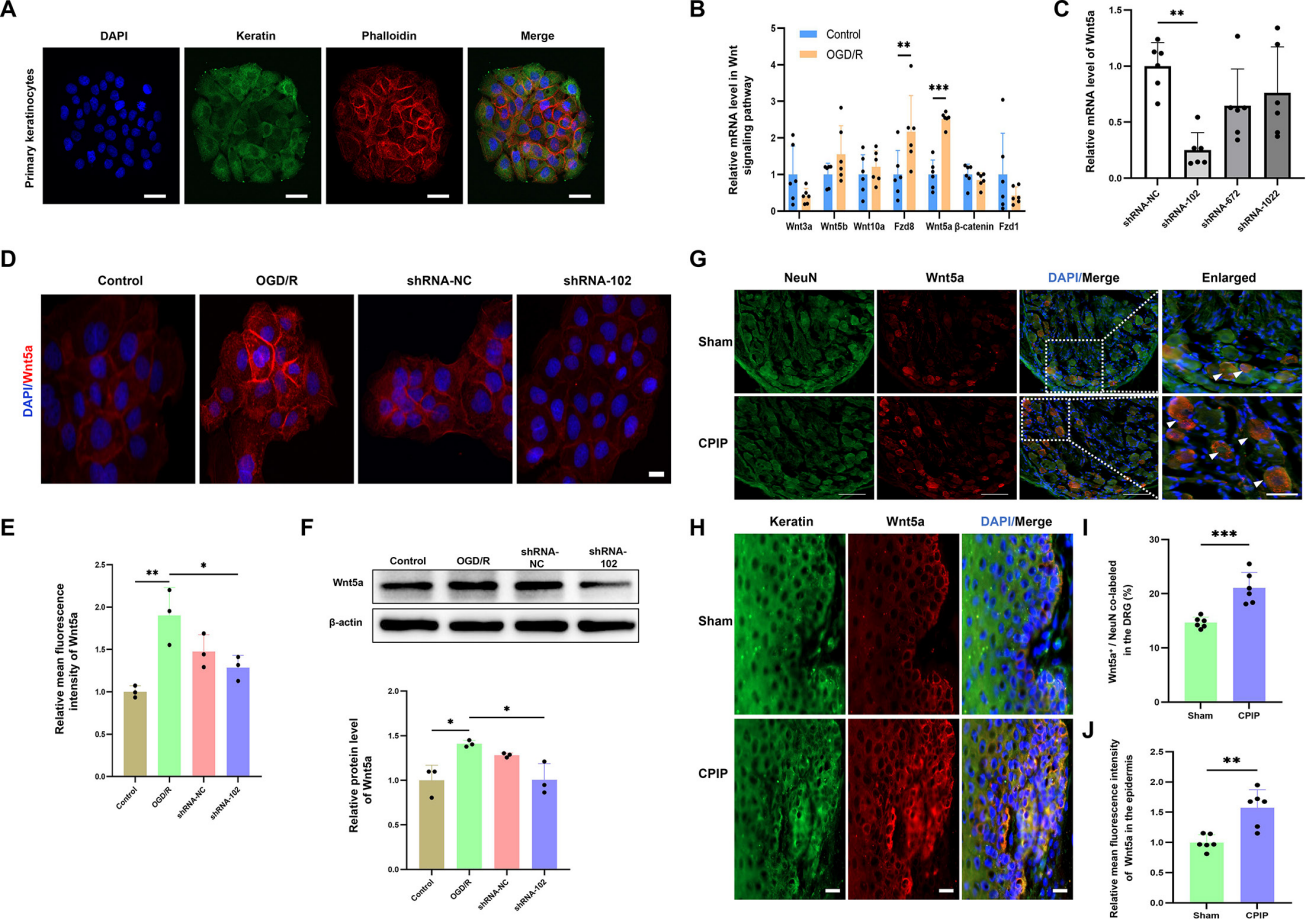
Expression of Wnt5a in primary keratinocytes and skin. (A) Combined keratin (a specific marker of keratinocytes) and phalloidin (a specific marker of F-actin) immunofluorescence staining in primary keratinocytes. Scale bar, 50 µm. (B) mRNA level of Wnt signaling pathway in the control and OGD/R group. **p<0.01, ***p<0.001, n=6. (C) Validation of Wnt5a shRNA transfection efficiency in primary keratinocytes. F=6.838, **p<0.01, n=6. (D, E) Immunofluorescence of Wnt5a in keratinocytes. F=9.97, *p<0.05, **p<0.01, n=3. Scale bar, 20 µm. (F) Western blots of the effect of shRNA-102 on Wnt5a. F=7.992, *p<0.05, n=3. (G, I) Immunofluorescence of Wnt5a in the DRG. Scale bar, 200 µm. Dashed insets show a higher magnification of the ROI in the inlay. Scale bar, 50 µm. ***p<0.001, n=6. (H, J) Immunofluorescence of Wnt5a in the epidermis. Scale bar, 20 µm. **p<0.01, n=6. DRG, dorsal root ganglion; OGD/R, oxygen-glucose deprivation/reoxygenation; ROI, region of interest.

We first isolated primary keratinocytes and identified them using the specific marker keratin (figure 1A). The results from qPCR revealed that Wnt5a expression was increased significantly after OGD/R treatment (p<0.001, figure 1B). To further verify the function of Wnt5a, lentivirus-carried shRNA-Wnt5a (shRNA102, shRNA672, and shRNA1022) were used to knock down Wnt5a. Compared with shRNA-negative control (NC), the qPCR results showed that the knockdown was most effective with shRNA-102 (p=0.001, figure 1C). Therefore, shRNA-102 was used for subsequent experiments.

Our findings show that the fluorescence intensity (p=0.003, figure 1D,E) and the protein level (p=0.017, figure 1F) of Wnt5a increased significantly in the OGD/R group, both of which were inhibited by shRNA-102. In rats, the expression of Wnt5a in the DRG (figure 1G,I) and epidermis (figure 1H,J) also increased significantly in vivo in the CPIP group.

### Knockdown of Wnt5a inhibited the upregulations of NR2B and MMP9

**Figure 2:**
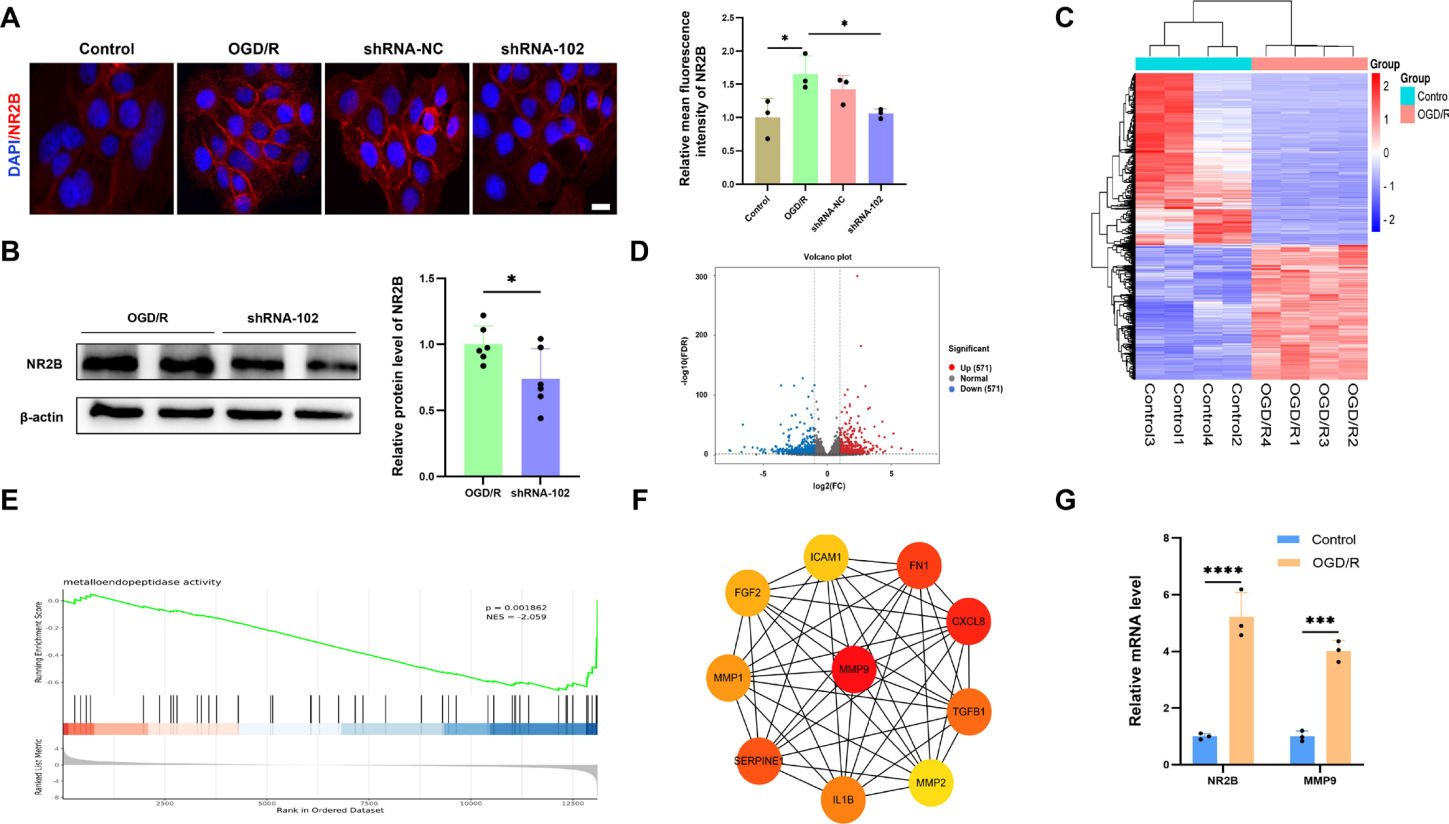
Knockdown of Wnt5a inhibited the upregulations of NR2B and MMP9 induced by OGD/R. (A) Immunofluorescence of NR2B in keratinocytes. Scale bar, 20 µm. F=5.729, *p<0.05, n=3. (B) Western blots of the protein level of NR2B. *p<0.05, n=6. (C, D) A heatmap and volcano plot based on RNA-seq analysis between the control and OGD/R group. (E) Enrichment plot for metalloendopeptidase activity by GSEA analysis. (F) PPI network analysis based on DEGs. (G) mRNA level of NR2B and MMP9 in the control or OGD/R group. ***p<0.001, ****p<0.0001, n=3. DEGs, differentially expressed genes; GSEA, Gene Set Enrichment Analysis; OGD/R, oxygen-glucose deprivation/reoxygenation; PPI, protein–protein interaction.

Our previous study showed that NR2B is expressed in keratinocytes which can be activated by NMDA,^9^ but the mechanism of NR2B activation remains unclear. In this setting, the upregulation of NR2B in keratinocytes after OGD/R treatment was inhibited by shRNA-102 (p=0.047, figure 2A). The expression of phosphorylated NR2B in keratinocytes is shown in online supplemental figure S3. As expected, the knockdown of Wnt5a prevented the increase of NR2B expression induced by OGD/R (p=0.038, figure 2B).

To further examine the hub genes associated with CRPS, HaCaT cells were used for RNA-seq analysis (figure 2C,D). GSEA analysis identified one significant enriched pathway: metalloendopeptidase activity (figure 2E). PPI analysis suggested that MMP9 may be an important contributor to the pathophysiology of CRPS (figure 2F). Moreover, qPCR analysis revealed that NR2B and MMP9 mRNA levels increased significantly after OGD/R treatment (figure 2G), and Wnt5a knockdown reduced the upregulation of MMP9 (online supplemental figure S4).

### Mitochondrial impairment and inflammation regulated by Wnt5a

**Figure 3:**
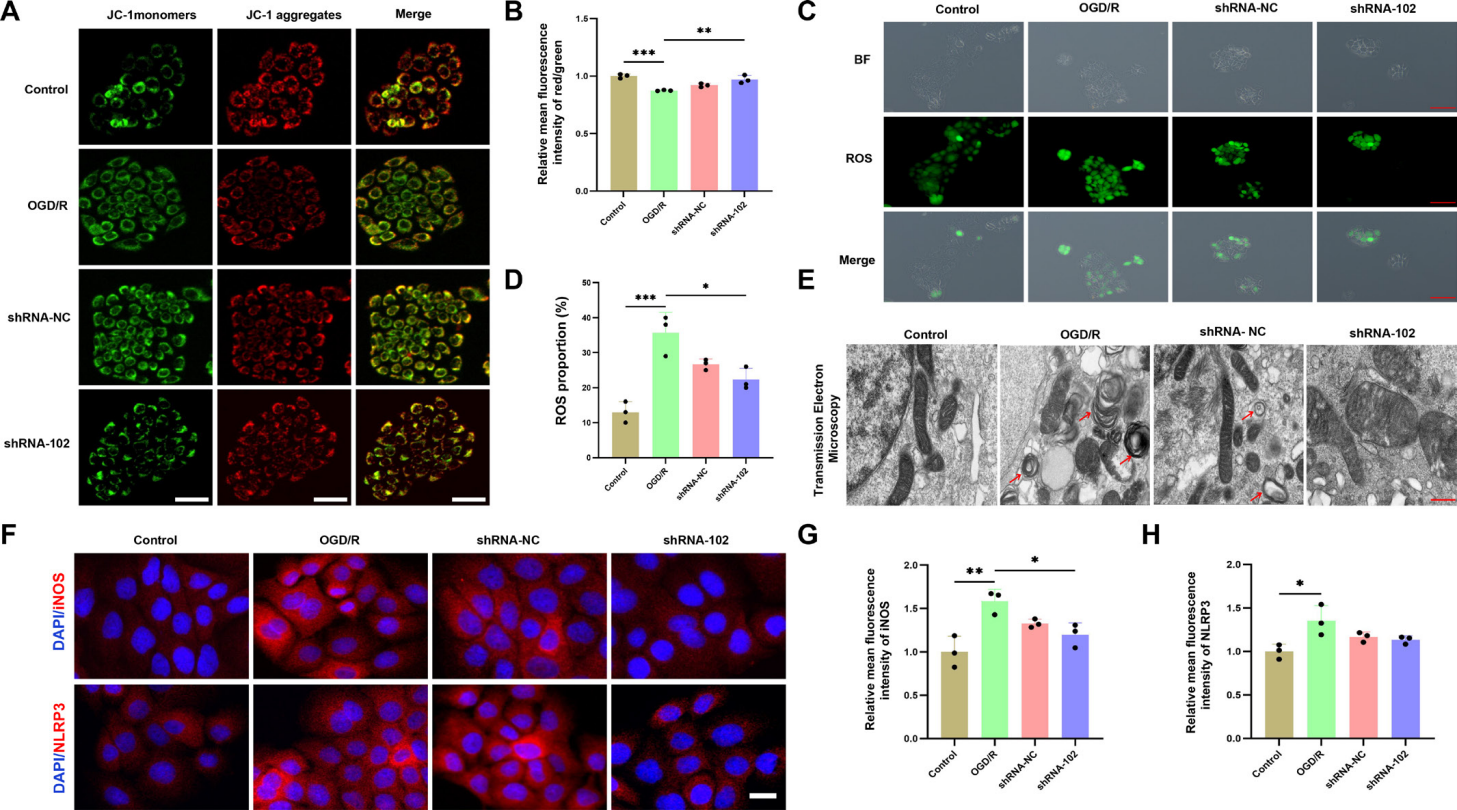
Mitochondrial impairment and inflammation regulated by Wnt5a. (A) Mitochondrial membrane potential assayed by JC-1 staining in keratinocytes. Scale bar, 100 µm. (B) Quantitative analysis of the JC-1 aggregate-to-monomer ratio (red/green). F=20.37, **p<0.01, ***p<0.001, n=3. (C) Intracellular ROS level of keratinocytes. Scale bar, 100 µm. (D) Quantitative analysis of ROS proportion in keratinocytes. F=19.02, *p<0.05, ***p<0.001, n=3. (E) Mitochondria morphological changes in keratinocytes by transmission electron microscopy. Red arrows illustrate shrunken and impaired mitochondria. Scale bar, 500 nm. (F) Immunofluorescence of iNOS and NLRP3 in keratinocytes. Scale bar, 20 µm. (G) Quantitative analysis of fluorescence intensity of iNOS. F=10.04, *p<0.05, **p<0.01, n=3. (H) Quantitative analysis of fluorescence intensity of NLRP3. F=5.938, *p<0.05, n=3. iNOS, inducible nitric oxide synthase; ROS, reactive oxygen species.

Mitochondria play an essential role in oxidative stress.^19^ Our findings show that the mitochondrial membrane potential was decreased significantly after OGD/R (p<0.001). Importantly, treatment with shRNA-102 attenuated this change (p=0.003, figure 3A,B).

ROS may induce mitochondrial impairment, and we found that increased ROS levels were attenuated by knocking down Wnt5a (p=0.010, figure 3C,D). Transmission Electron Microscopy images revealed increased mitochondrial fragmentation and cristae vacuolation following OGD/R, with phagophores enwrapping the cytoplasmic components and maturing into double-membrane vesicles (figure 3E). Inducible nitric oxide synthase (iNOS) and the activation of NOD-like receptor thermal protein domain associated protein 3 (NLRP3) in peripheral tissues may be involved in neuropathic pain. In the current study, inhibition of Wnt5a also suppressed the upregulation of iNOS (p=0.031, figure 3F,G) but not NLRP3 (p=0.119, figure 3F,H).

### Wnt5a inhibition attenuates CRPS-related pain behavior

**Figure 4:**
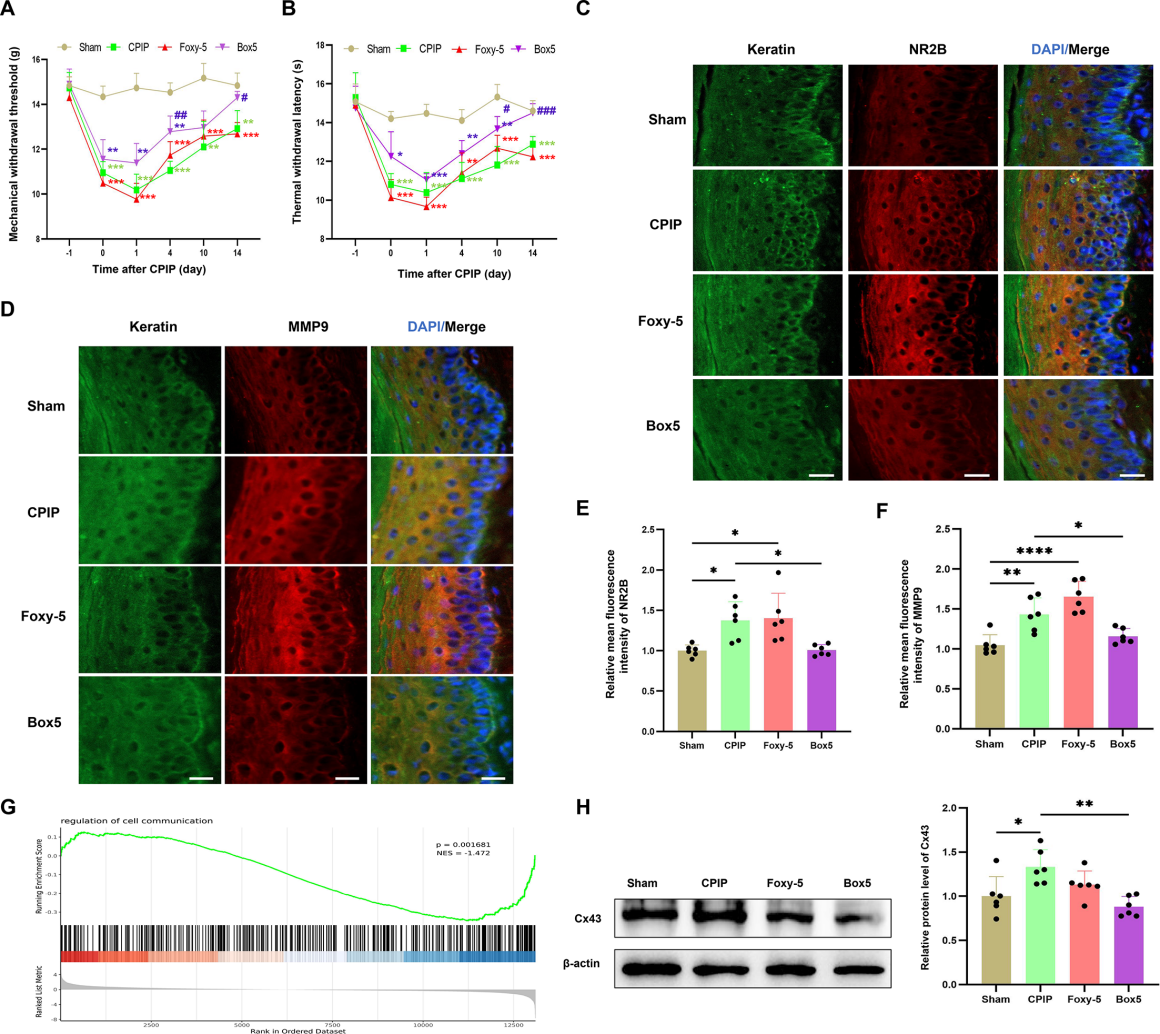
Wnt5a inhibition attenuates pain behavior and inflammatory response induced by CPIP. (A, B) Effect of Foxy-5 and Box5 on the development of CPIP-induced mechanical allodynia (A) and thermal hyperalgesia (B). *p<0.05, **p<0.01, ***p<0.001, compared with Sham group, #p<0.05, ##p<0.01, ###p<0.001, compared with the CPIP group, n=6. (C, D) Immunofluorescence of NR2B and MMP9 in the epidermis. Scale bar, 20 µm. (E) Quantitative analysis of fluorescence intensity of NR2B. F=7.531, *p<0.05, n=6. (F) Quantitative analysis of fluorescence intensity of MMP9. F=16.94, *p<0.05, **p<0.01, ****p<0.0001, n=6. (G) Enrichment plot for regulation of cell communication by GSEA analysis. (H) Western blots of the effect of Wnt5a on Cx43 induced by CPIP. F=7.091, *p<0.05, **p<0.01, n=6. CPIP, chronic post-ischemia pain; GSEA, Gene Set Enrichment Analysis.

Behaviorally, the subcutaneous injection of Box5 mitigated the decreased MWT (p=0.003, day 4; p=0.026, day 14) and TWL in CPIP rats (p=0.015, day 10; p<0.001, day 14, figure 4A,B). The expressions of NR2B and MMP9 were increased significantly in the CPIP group, and Box5 treatment significantly attenuated the upregulated NR2B (p=0.021) and MMP9 (p=0.040, figure 4C–F). These findings suggest that Wnt5a-mediated NR2B/MMP9 activation may be an important mechanism in triggering pain sensitization after CPIP.

Based on GSEA analysis, changes in cell communication may contribute to CRPS (figure 4G). Increasing evidence indicates the critical role of connexins in the pathogenesis of neuropathic pain, especially Cx43, which can form hemichannels mediating the communication between keratinocytes.^5 20^ Western blotting analysis shows that CPIP-induced overexpression of Cx43 was inhibited by Box5 (p=0.002, figure 4H). Similarly, immunofluorescence analysis also suggested that Box5 inhibited the overexpression of Cx43 (online supplemental figure S5). ELISA analysis indicates a significant inflammatory response in the epidermis after CPIP, and Box5 alleviated regional inflammation, compared with Foxy-5 control (online supplemental figure S6).

### NR2B and MMP9 activation in the DRG contributed to peripheral pain sensitization

**Figure 5:**
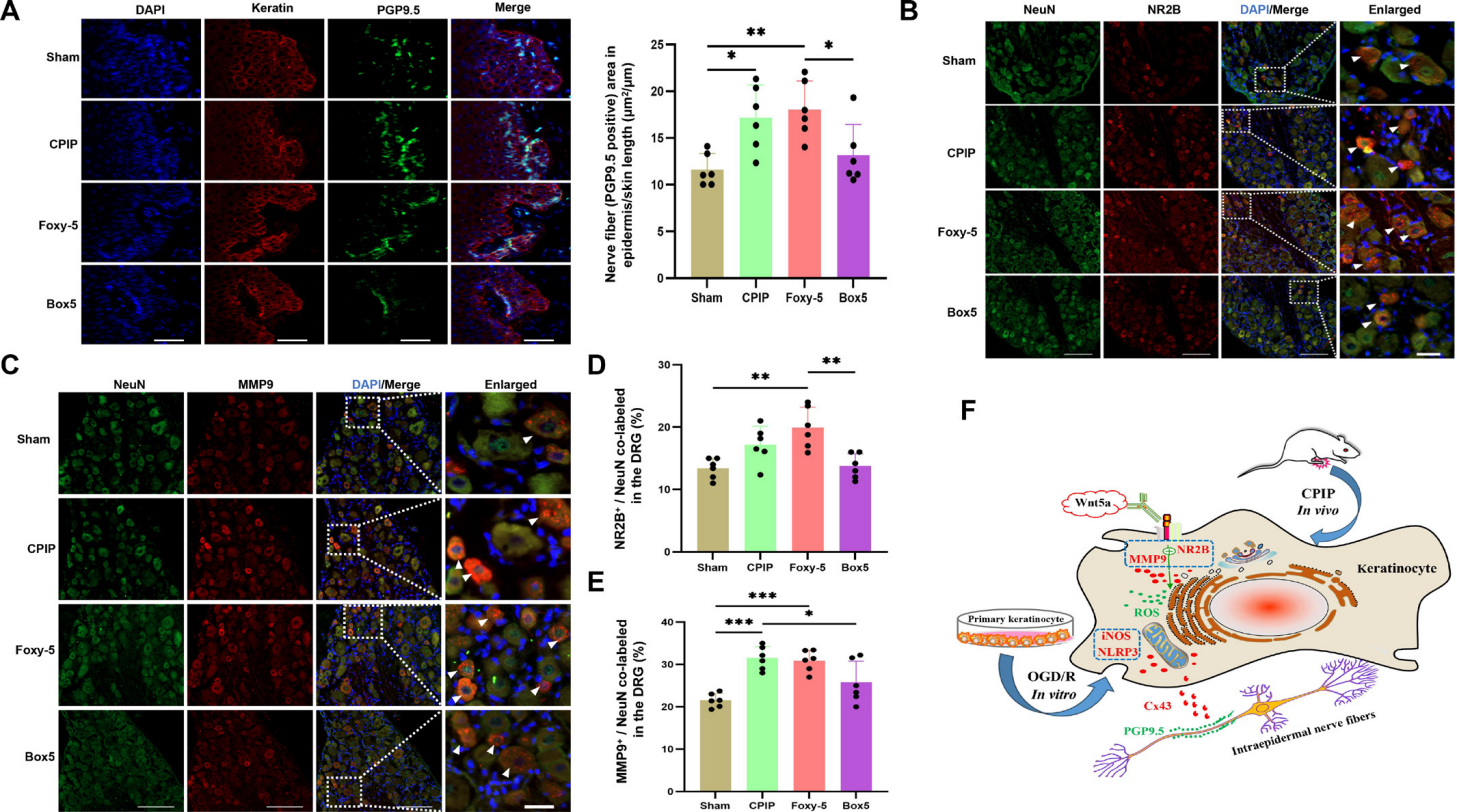
NR2B and MMP9 activation in the DRG contributed to peripheral sensitization. (A) Immunofluorescence of PGP9.5 in the epidermis. Scale bar, 50 µm. F=6.549, *p<0.05, **p<0.01, n=6. (B, C) Immunofluorescence of NR2B (B) and MMP9 (C) co-labeled with NeuN in the DRG. Scale bar, 200 µm. Dashed insets show a higher magnification of the ROI in the inlay. Scale bar, 50 µm. (D) Quantitative analysis of NR2B^+^/NeuN (%) co-labeled in the DRG. F=8.624, **p<0.01, n=6. (E) Quantitative analysis of MMP9^+^/NeuN (%) co-labeled in the DRG. F=12.60, *p<0.05, ***p<0.001, n=6. (F) Model for Wnt5a in keratinocytes driving peripheral sensitization. CRPS, complex regional pain syndromel; DRG, dorsal root ganglion; ROI, region of interest.

The dysfunction of intraepidermal nerve fiber and DRG neurons may contribute to the development of chronic pain.^5 21^ Here, we found that the fluorescence intensity of nerve fiber labeled with PGP9.5 increased significantly after CPIP (p=0.020). This upregulation was inhibited by Box5, compared with the Foxy-5 group, but not with the CPIP group (p=0.045, figure 5A). Compared with the Sham group, NR2B co-labeled with NeuN was significantly increased only in the Foxy-5 group (p=0.002, figure 5B,D). MMP9 showed a statistical difference in both CPIP (p=0.012) and Foxy-5 groups (p=0.001, figure 5C,E).

## Discussion

CRPS is a chronic pain condition with a magnitude disproportionate to the expected pain experienced after trauma.^22^ Given the current lack of effective treatment for CRPS, identifying new therapeutic targets is important. Skin keratinocytes can elicit the transmission of nociceptive signals through intrinsic sensory transduction mechanisms.^4 23^ However, the contribution of keratinocytes to the pathophysiology of CRPS remains unclear. Our findings suggest that Wnt5a in skin keratinocytes may play an important role in CRPS via the activation of downstream NR2B and MMP9.

The aberrant activation of the Wnt signaling pathways may contribute to chronic pain by enhancing inflammation. In line with this notion, we observed a significant increase of Wnt5a in keratinocytes following in vitro OGD/R treatment. Similarly, MMP9 shows a rapid and transient upregulation in the injured DRG, consistent with findings observed in the early phase of neuropathic pain.^24^ This upregulation may be due to interleukin-1β cleavage and microglia activation induced by MMP9.

Mitochondria maintain the dynamic balance in keratinocytes through fission, fusion, and autophagy. Our study showed that the excessive accumulation of ROS led to a decrease in mitochondrial membrane potential, suggesting mitochondrial dysfunction may be a key factor underlying pain sensitization associated with CRPS. The iNOS and NLRP3 inflammasome, which are correlated with the ROS pathway, are indicators of cellular ‘danger’.^25 26^ However, only the overexpression of iNOS was inhibited by Wnt5a knockdown, indicating their different roles in sensing mitochondrial dysfunction.

CRPS is characterized by allodynia and hyperalgesia after ischemia-reperfusion of the skin.^27^ Our findings showed that Box5 significantly attenuated CRPS-related pain behavior on day 14. Cx43 is abundantly expressed in the keratinocytes, allowing secondary messengers to be involved in intercellular communication.^28^ We further identified Cx43 in close proximity to nerve fibers as a potential contributor to pain sensitization. A notable increase of Cx43 was also observed in the DRG and spinal cord in peripheral nerve injury models.^20^ Additionally, changes in PGP9.5 expression may correlate with the development of neuropathic pain,^29^ which is consistent with our findings.

Targeting DRG neurons may effectively alleviate pain.^1^ We observed an upregulation of NR2B and MMP9 levels in the lumbar DRGs in CPIP rats. Importantly, Box5 attenuated the upregulated MMP9. These findings reveal the previously unidentified contribution of MMP9 in the DRG to pain sensitization and suggest that the application of Wnt5a inhibitors, such as Box5, to the skin may present a new treatment for CRPS. It should be acknowledged that our in vivo study was performed only at the chronic phase of CRPS (day 14). Further studies need to evaluate the role of Wnt5a at the acute phase of CRPS (<24 hours). Additionally, ultrastructural morphological and electrophysiological studies are needed to further explore the mechanisms of keratinocyte-nerve fiber interaction.

## Conclusions

Our findings suggest that Wnt5a in skin keratinocytes may play an important role in CRPS, presumably by activating NR2B and MMP9 to induce peripheral pain sensitization. Studying the interaction between keratinocytes and nerve fibers will help to better understand the mechanism of CRPS and provide new perspectives on its management (figure 5F).

## Supplementary material

10.1136/rapm-2024-106139 online supplemental file 1

## Data Availability

Data are available on reasonable request.
