# Supplementary material for: Wnt5a in keratinocytes contributes to complex regional pain syndrome through the activation of NR2B and MMP9 in rats
Source: Reg Anesth Pain Med. 2025 Mar 12;51(7):e106139. doi: 10.1136/rapm-2024-106139 (PMC13422098; doi:10.1136/rapm-2024-106139)
Supplement: online supplemental file 1 [file rapm-51-7-s001.pdf]

Supplementary materials

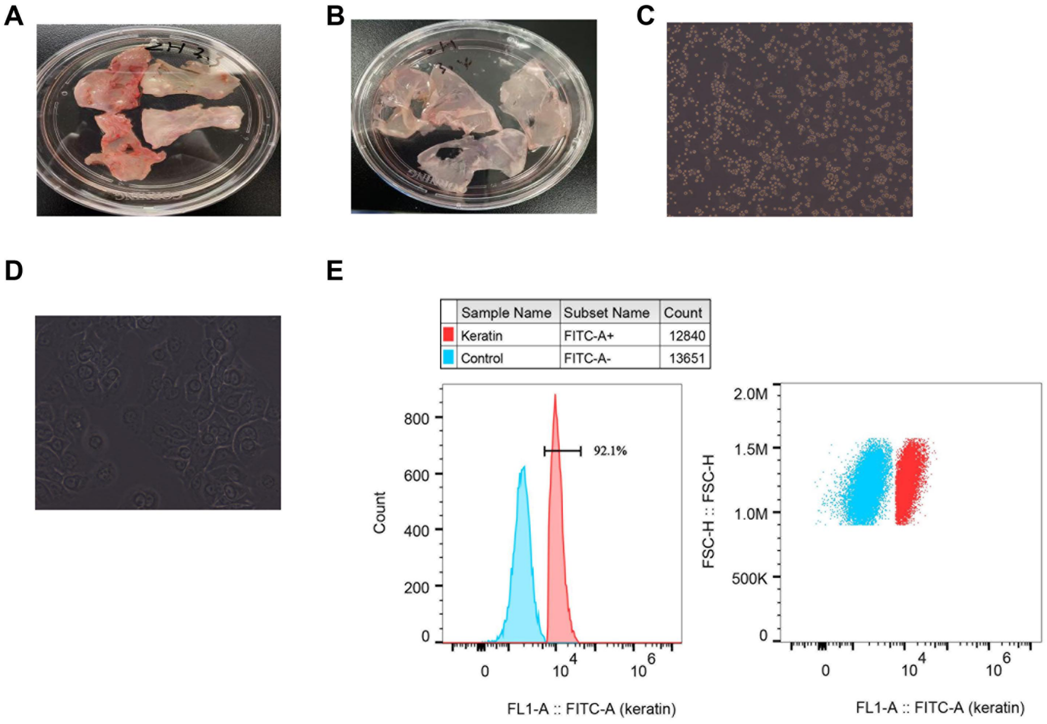

Figure S1. Primary keratinocytes isolation and culture. (**A**) Sacrifice the post-natal day 0-2 neonates from the Sprague–Dawley rat strain by decapitation using scissors then peeling off the whole skin and acquired epidermis. (**B**) The epidermis was fully infiltrated with 0.3% Dispase II (Sigma, USA) for 24 hours at 4°C, then gently scraped off the dermis to obtain a complete epidermis. (**C**) The epidermis was fully infiltrated with 0.05% Trypsin-EDTA (Gibco, USA) for 20 minutes at 37°C to obtain keratinocytes. (**D**) The keratinocytes were transferred to cell culture dish containing EpiLife medium and cultured at a temperature of 37°C under a controlled atmosphere of 5% CO2. **(E)** Flow cytometry histograms showing the specificity keratin antibody for primary keratinocytes.

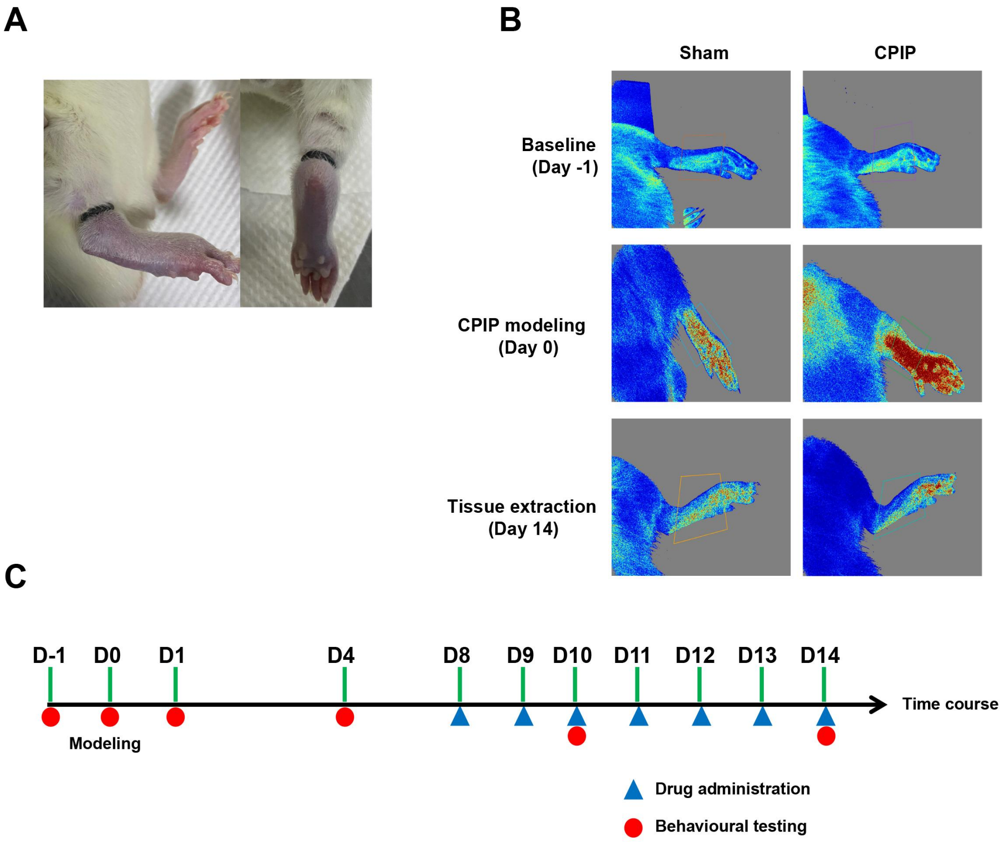

Figure S2. Modeling and drug administration flow chart. (**A**) Adult Sprague–Dawley rats were anesthetized via intraperitoneal injection of 60 mg/kg sodium pentobarbital. Then, a Nitrile 70 Durometer O-ring with an internal diameter of 0.56 cm (Kangda Chemical Company, Shanghai, China) was placed around the right hind limb for 3 hours. The Sham group rats were loosely surrounded by cut O-rings. (**B**) The blood perfusion of the right hind limb was detected by Doppler flowmeter in different time periods. (**C**) Flow chart of rat drug administration and behavioral testing, the D0 behavioral measurement was made 6 hours after modeling.

Table S1 List of the target sequence of shRNA

NO	TargetSeq
shRNA102	5′-CTCCTTCGCCCAGGTTGTTAT-3′
shRNA672	5′-AGCAGGCCGTAGGACAGTATA-3′
shRNA1022	5′-GGCGTGGCTATGACCAGTTTA-3′
shRNA- NC	5′-TTCTCCGAACGTGTCACGT-3′

Table S2 List of the primer sequence for RT-qPCR

Target	Direction	Primer sequence 5′-3′	Product size (bp)
Wnt3a	Forward	GTGTCAAGGCGGGCATCCAAG	150
	Reverse	CAGCGGAAGCGATGGCATGG	
Wnt5b	Forward	CGTGGAGTATGGCTACCGCTTTG	87
	Reverse	CCCTGCTCCTCTGATCCCTTGG	
Wnt10a	Forward	GAGTGCTTTCGCCTACGCCATAG	94
	Reverse	ATCGCAACCGCAAGCCTTCAG	
Frizzled-8	Forward	CAACCAGAGCCTTGACAACCTACG	123
(Fzd8)	Reverse	TTGATGACTGAGCGGATTCGGAAC	
Wnt5a	Forward	CAACTGGCGGGACTTTCTCAAGG	116
	Reverse	CGGAACTGGTACTGGCACTCTTTG	
β-catenin	Forward	CCGTTCGCCTTCATTATGGACTACC	113
	Reverse	TGGGCAAAGGGCAAGGTTTCG	
Frizzled-1	Forward	GCATGACGGCACCAAGACAGAG	106
(Fzd1)	Reverse	GAAGTAGCAGGCGATGACGATGG	
NR2B	Forward	TTTCCATCGTCACCACCTACTTCC	140

	Reverse	CTTAGAGTCGCCATCGTCCAGAG	
MMP9	Forward	ACCGCCAACTATGACCAGGATAAG	122
	Reverse	TGCTTGCCCAGGAAGACGAAG	
β-actin	Forward	GCTGTGCTATGTTGCCCTAGACTTC	122
	Reverse	GGAACCGCTCATTGCCGATAGTG	

Table S3 Antibodies used for western blot and immunofluorescence.

Antibody	Host	Company	Catalog number	Usage	Dilution
Keratin	Mouse	Abcam	ab8068	IF	1:100
	Rabbit	Proteintech	10712-1-AP	IF	1:200
Phalloidin	Rabbit	Proteintech	PF00003	IF	1:500
Wnt5a	Rabbit	Bioss	Bs-1948R	WB	1:1000
				IF	1:200
NR2B	Rabbit	Proteintech	21920-1-AP	WB	1:1000
				IF	1:200
pNR2B	Rabbit	Abcam	Ab18532	IF	1:200
MMP9	Rabbit	Abcam	Ab283575	IF	1:500
iNOS	Rabbit	Proteintech	18985-1-AP	IF	1:200
NLRP3	Rabbit	Invitrogen	SC-06-23	IF	1:1000
Cx43	Rabbit	Proteintech	26980-1-AP	WB	1:200
				IF	1:200
PGP9.5	Mouse	Proteintech	66230-1-Ig	IF	1:100
NeuN	Mouse	Proteintech	66836-1-Ig	IF	1:100
β-actin	Mouse	Proteintech	66009-1-Ig	WB	1:5000

Anti-Rabbit IgG HRP	Goat	Proteintech	SA00001-2	WB	1:5000
Anti-Mouse IgG HRP	Goat	Proteintech	SA00001-1	WB	1:5000
488-conjugated Anti-Mouse IgG	Goat	Proteintech	SA00013-1	IF	1:500
594-conjugated Anti-Rabbit IgG	Goat	Proteintech	SA00013-4	IF	1:500

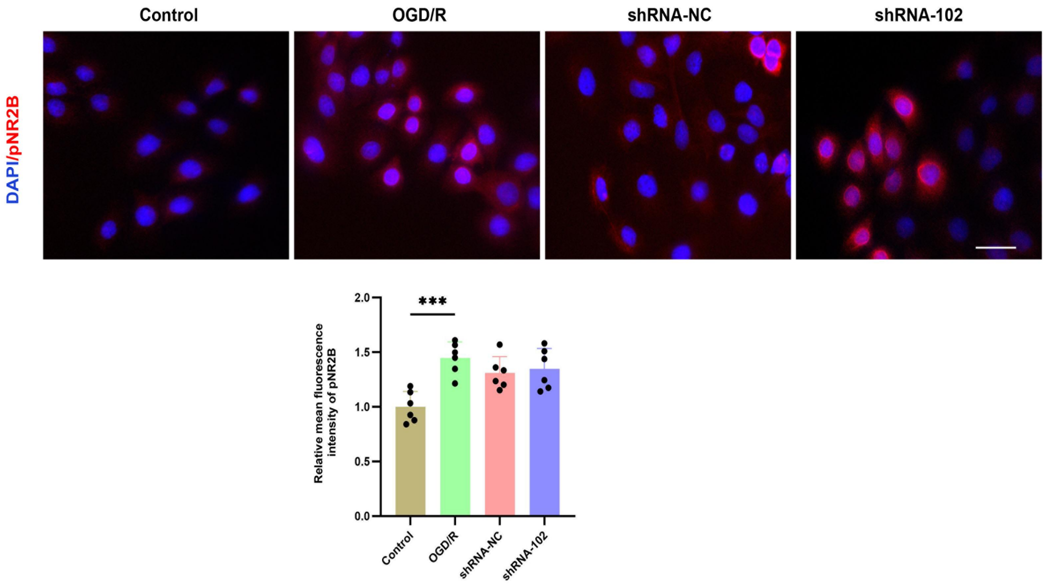

Figure S3. Representative immunofluorescence staining of pNR2B in keratinocytes. F=9.081, ***P<0.001, n=6. Scale bar, 50 μm.

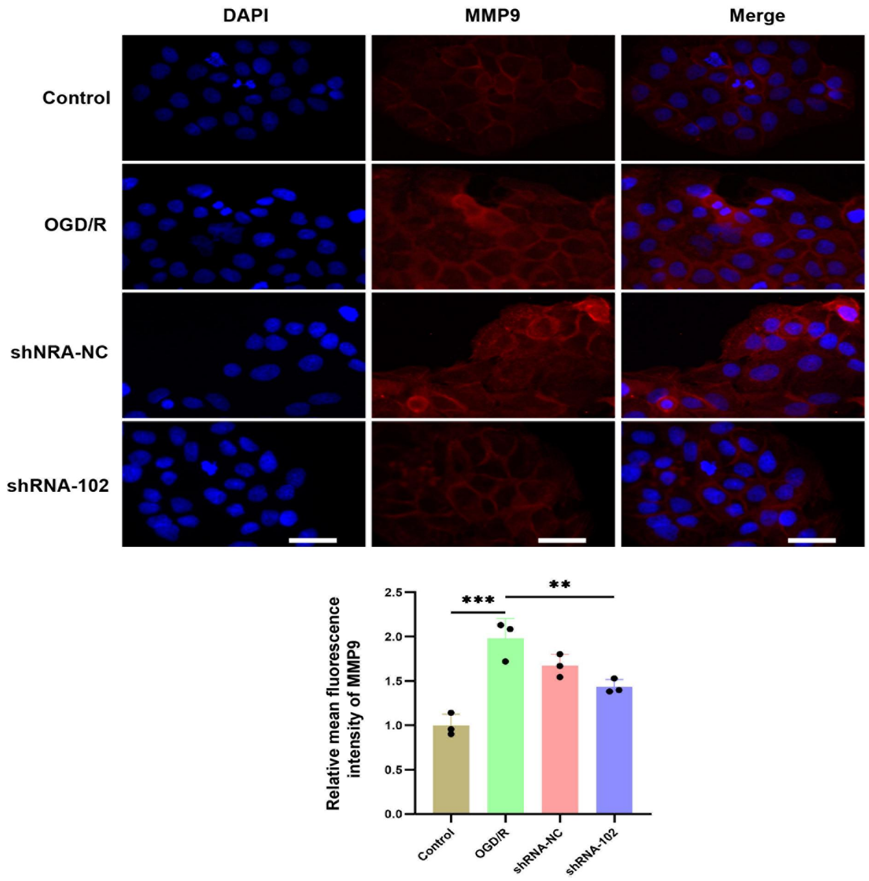

Figure S4. Knockdown of Wnt5a inhibited the increased expression MMP9 induced by OGD/R. Representative immunofluorescence staining of MMP9 in the keratinocytes. Scale bar, 50 μm. F=22.76, $^{**}P<0.01$, $^{***}P<0.001$, n=3.

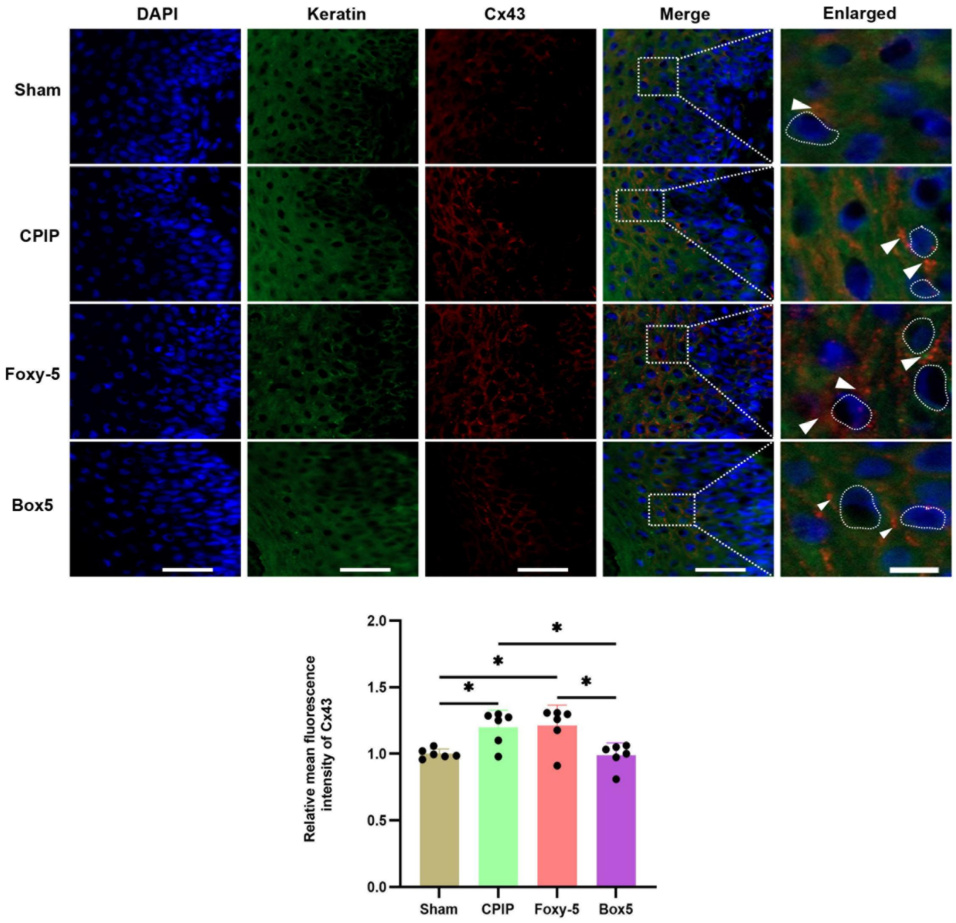

Figure S5. Representative immunofluorescence staining of Cx43 in the epidermis. Scale bar, 50 μm. Dashed insets show higher magnification of the ROI in inlay and arrow indicates Cx43 plaques located between keratinocytes. Scale bar, 10 μm. F=6.989, *P<0.05, n=6.

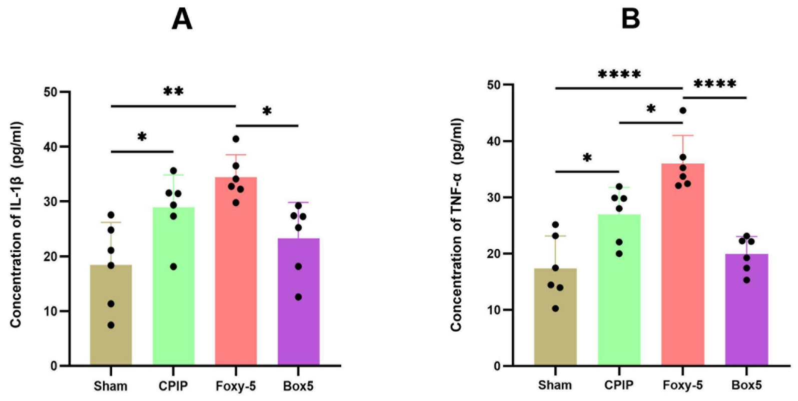

Figure S6. (**A**) Concentration of IL-1β analyzed by ELISA assay in the epidermis. F=7.455, *P<0.05, **P<0.01, n=6. (**B**) Concentration of TNF-α analyzed by ELISA assay in epidermis. F=18.45, *P<0.05, ****P<0.0001, n=6.
